# Oxidant/Antioxidant Imbalance in Alzheimer's Disease: Therapeutic and Diagnostic Prospects

**DOI:** 10.1155/2018/6435861

**Published:** 2018-01-31

**Authors:** Joanna Wojsiat, Katarzyna Marta Zoltowska, Katarzyna Laskowska-Kaszub, Urszula Wojda

**Affiliations:** Laboratory of Preclinical Testing of Higher Standard, Nencki Institute of Experimental Biology, Polish Academy of Science, Pasteur 3 St., 02-093 Warsaw, Poland

## Abstract

Alzheimer's disease (AD) is the most common cause of dementia and a great socioeconomic burden in the aging society. Compelling evidence demonstrates that molecular change characteristics for AD, such as oxidative stress and amyloid *β* (A*β*) oligomerization, precede by decades the onset of clinical dementia and that the disease represents a biological and clinical continuum of stages, from asymptomatic to severely impaired. Nevertheless, the sequence of the early molecular alterations and the interplay between them are incompletely understood. This review presents current knowledge about the oxidative stress-induced impairments and compromised oxidative stress defense mechanisms in AD brain and the cross-talk between various pathophysiological insults, with the focus on excessive reactive oxygen species (ROS) generation and A*β* overproduction at the early stages of the disease. Prospects for AD therapies targeting oxidant/antioxidant imbalance are being discussed, as well as for the development of novel oxidative stress-related, blood-based biomarkers for early, noninvasive AD diagnostics.

## 1. Complex Pathomechanisms of Alzheimer's Disease

Alzheimer's disease (AD) is a devastating, progressive neurodegenerative disorder presenting with cognitive decline and memory impairments. It can be best described as a biological and clinical continuum, covering preclinical (presymptomatic) and clinical (symptomatic) phases, with the latter traditionally subcategorized into mild cognitive impairment (MCI) due to AD (prodromal AD) and AD dementia with mild, moderate, and finally severe late stages [1, 2].

Neuropathological hallmarks of the disease are extracellular amyloid plaques and intracellular neurofibrillary tangles accumulating in the brains of AD patients [3]. Besides these AD-specific features, a large body of evidence indicates the presence of a number of other pathologies which are common for multiple neurodegenerative diseases and for normal brain aging, reviewed in [4]. These include prominent activation of inflammatory and innate immune responses as well as excessive iron deposition and mitochondrial damage [5].

The etiology of AD is incompletely understood. Most of the AD cases are late-onset, sporadic (SAD), with probably very complex origin, influenced by genetic background [6, 7], environmental exposures [8], and aging, with age being the greatest risk factor [9, 10]. Apolipoprotein E (ApoE) genotype is the second risk factor for AD after age. The ApoE exists in three isoforms: ApoE2, ApoE3, and ApoE4, with the ApoE3 being the most common variant. The risk for AD is increased 2-3-fold in the ApoE4 heterozygous carriers and 12-fold in the homozygous ones, and each ApoE4 allele lowers the AD age of onset by approximately 5 years. On the contrary, the ApoE2 isoform appears to protect against the disease [11–14].

Despite most AD cases being sporadic, 1% of cases represent an early-onset, familial subtype of the disease (FAD), which is caused by mutations in the amyloid precursor protein *(APP)* [15], presenilin 1 (*PSEN1*) [16], and presenilin 2 (*PSEN2*) [17] genes. Amyloidogenic processing of APP by *β*-secretase (BACE) and next by *γ*-secretase, the catalytic component of which is presenilin (PS), results in the generation of a wide spectrum of amyloid *β* (A*β*) peptides [18–21], with the longer forms being the most toxic, prone to aggregation and deposition in the form of amyloid plaques in the brain [2, 22, 23].

The detection of amyloid plaques in the brains of AD patients and the discovery of pathogenic mutations in *APP* and *PSEN1* genes have led to the formulation of the amyloid cascade hypothesis, which proposes that A*β* is the main culprit in AD pathogenesis [24]. This hypothesis was later supported by the data indicating that the A673T substitution in APP, located adjacent to the BACE1 cleavage site and making the APP a less favorable substrate for BACE1, protects against cognitive decline and AD [25]. Nevertheless, further studies have demonstrated that although A*β* deposition is necessary for the development of the disease it is not sufficient, since the A*β* pathology can exist with the lack of clinical dementia [26]. In addition, several clinical trials targeting A*β* failed to provide therapeutic benefits [27]. These findings have led to the refinement of the amyloid cascade hypothesis and to the formulation of several new hypotheses, including the mitochondrial cascade and oxidative stress hypotheses, which place AD in the context of a free radical theory of aging [28–31], and the dual pathway hypothesis, which proposes the existence of common upstream drivers that cause amyloid and tau pathologies [32].

**Figure 1:**
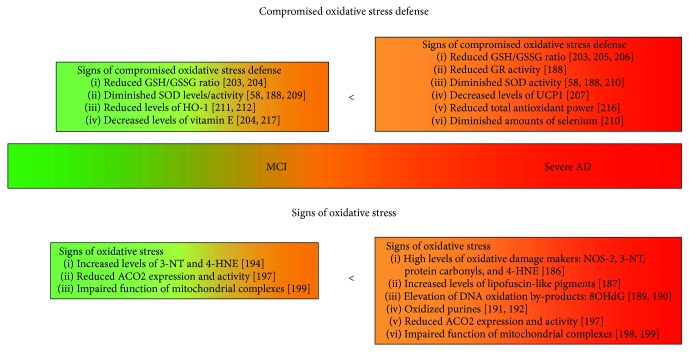
Escalating signs of the antioxidant/oxidant imbalance detected in whole blood during the AD progression. The figure schematically presents the progressive impairments in the oxidative stress defense mechanisms during the progression from mild cognitive impairment (MCI) towards severe AD. These correspond to the elevation in the oxidative stress markers. The green-orange-red colour scale corresponds to healthy stage-MCI-AD progression. AD: Alzheimer's disease; MCI: mild cognitive impairment; ACO2: aconitase 2; GR: glutathione reductase; GSH/GSSG: reduced/oxidized glutathione pool; 4-HNE: 4-hydroxynonenal; NOS-2: nitric oxide synthase 2; 3-NT: 3-nitrotyrosine; 8OHdG: 8-oxo-2′-deoxyguanosine; SOD: superoxide dismutase; UCP-1: uncoupling protein 1.

Here, we will review the current knowledge on the implication of one of those possible drivers—oxidative stress—in the development of AD pathology (Figure 1), with the major emphasis placed on the interplay between reactive oxygen species (ROS) and A*β* deposition. Furthermore, we will summarize how oxidant/antioxidant imbalance, detected in the AD brain, is reflected in peripheral cells, pointing towards the potential development of novel, noninvasive, blood-based biomarkers for early AD diagnostics and for monitoring of the response to therapeutic interventions.

## 2. The Definition and Causes of the Oxidative Stress

Oxidative stress is defined as an imbalance between oxidants and antioxidants, in favor of the oxidants. It leads to the disruption of redox signaling and control and/or causes damage to biomolecules [33–35]. The oxidative stress is characterized by an excessive accumulation of toxic molecules containing an oxygen atom with an unpaired electron in its outer shell, formed as end products of the normal metabolism of oxygen and known as ROS. The main source of ROS is the electron transport chain (ETC) at the mitochondrial inner membrane, where energy is generated in the form of ATP [36]. Under physiological redox conditions in the cell, ROS fulfill important roles (redox biology) in cell signaling, homeostasis, and in the immune system's response against invading pathogens. In addition, reactive oxygen and nitrogen species regulate such key biological processes at the organismal level as blood circulation, energy metabolism, reproduction, embryonic development, and remodeling of tissues through apoptotic mechanism. Finally, it has been proposed that moderate levels of ROS may induce several protective responses that prepare cells to better handle the subsequent stress. This concept is known as mitohormesis [37–40].

Given the physiological importance of the moderate ROS levels and the harmful consequences of excessive ROS generation, precise mechanisms must have been developed to regulate reducing and oxidizing conditions within the cell (cellular redox state). Aerobic organisms have integrated antioxidant systems, which include enzymatic and nonenzymatic antioxidants, usually effective in scavenging ROS and preventing their detrimental consequences. Overwhelming and/or failure of the redox control and antioxidant systems would inevitably lead to the oxidative stress.

Neurons in the brain are at extremely high risk of excessive ROS generation and oxidative damage since they show high oxygen consumption and energy production. About 20% of all organismal oxygen and 25% of all glucose are used for cerebral functions [41]. The vulnerability of neurons to the oxidative damage stems also from a relatively large amount of redox active metals, promoting ROS formation, high content of polyunsaturated fatty acids (PUFA), which are more sensitive to oxidation, and from comparatively modest levels of antioxidant enzymes [42].

Given the vulnerability of neurons to ROS, it is not surprising that oxidative stress has been widely proposed to be implicated in the initiation and the progression of a number of neurodegenerative diseases, including AD [43–47]. In line with that, markers of oxidative damage [48] to (1) lipids, indexed by thiobarbituric acid reactive substances (TBARS), alterations in PUFA, and the presence of PUFA breakdown products [45, 49, 50]; (2) proteins, marked by protein carbonyls, 3-nitrotyrosine (3-NT), and 4-hydroxy-2-nonenal (4-HNE) [51, 52]; and (3) nucleic acids [53], including nuclear [54] and mitochondrial DNA [55–57] as well as RNA [45], marked by 8-hydroxy-2′-deoxyguanosine (8OHdG) and 8-hydroxyguanosine (8-OHG), have been frequently described in the brains of MCI and AD patients. Importantly, the oxidative damage present in the brain is reflected in peripheral cells, raising a possibility for further research and the potential use of the oxidative stress biomarkers for noninvasive blood-based AD diagnostic and monitoring [58–60]. However, no validated blood-based biomarker has been established so far.

## 3. Oxidative Stress in AD Brain: Interplay between ROS and A*β*

Although it has been widely accepted that the presence of oxidative damage is one of the pathological hallmarks of AD, it remains uncertain whether this is a cause or a consequence of pathogenic processes occurring in the brain. The hypothesis that oxidative damage is an early event in the disease, triggering the occurrence of other pathologies, is supported by the association of the AD risk factors, including ApoE4 genotype, with higher oxidative insults. Comparison of the hippocampi from AD patients demonstrated that ApoE protein expression is reduced in ApoE4 carriers compared to ApoE3/ApoE3 controls and indicated an association between decreased ApoE protein amounts and higher oxidative stress to lipids, manifesting with increased levels of TBARS [61]. Furthermore, levels of isoprostanes, markers of lipid peroxidation [62], have been reported to be increased in cerebrospinal fluid (CSF) in ApoE4-positive healthy individuals and in AD patients [63–66]. These observations are in line with *in vitro* data demonstrating that different ApoE isoforms possess different antioxidant properties, with ApoE2 being the most and ApoE4 the least protective variant [67].

Other well-established risk factors for AD are insulin resistance and type 2 diabetes mellitus. Several studies have demonstrated that oxidative stress plays an important role in the pathogenesis of these conditions and that there is an overlap between pathophysiological mechanisms implicated in type 2 diabetes and AD [68–73]. Hyperinsulinemia, often seen in type 2 diabetes, may inhibit degradation of extracellular A*β* through the insulin-degrading enzyme (IDE) [74]. Moreover, the hyperglycemia may result in enhanced metabolism of glucose and in turn in increased levels of NADH and FADH_2_, which are used by mitochondria to generate ATP. Overproduction of NADH leads to greater proton gradient and consequently to an excessive generation of ROS [71, 75].

In addition, the increased levels of intracellular glucose lead to the nonenzymatic glycosylation of cellular proteins, lipoproteins, or nucleic acids. The resulting products are known as advanced glycation end products (AGEs). RAGE is the receptor for AGE, and it is a pattern recognition receptor that binds multiple ligands derived from damaged cell environment [76] as well as A*β* [77]. The interaction of RAGE with its ligands results in enhanced ROS generation [78], due to the activation of NADPH oxidase (NOX) [79]. Of note, RAGE has been widely implicated in the pathomechanism of AD [80]. Increased expression of RAGE has been demonstrated in the brains of AD mouse models [81]. Administration of high-AGE diet has been found to lead to spatial memory impairments, oxidative damage to vasculature, and increased levels of insoluble A*β* in mouse hippocampi [82]. Finally, polymorphisms in RAGE encoding gene, *AGER*, have been reported to genetically predispose to AD [83]. Another link between diabetes mellitus and AD is evidenced by the fact that oxidative stress and insulin resistance may promote transcriptional activity of Forkhead box O (FoxO) proteins [84], resulting in enhanced expression of gluconeogenic enzymes, which leads to hyperglycemia and further increase in ROS production in a vicious cycle [85].

More indications of the early involvement of the oxidative stress in AD pathogenesis stem from the data showing that markers of oxidative stress in the APP23 mice, carrying APP KM670/671NL mutation, and triple transgenic mice, harboring APP KM670/671NL, PS1 M146 V, and Tau P301L mutations, are present relatively early, before the formation of amyloid deposits [86, 87]. Furthermore, analysis of A*β*40 production *in vitro* and *in vivo* upon pharmacological or genetic inhibition of mitochondrial ETC, either by targeting complex I and/or complex III, has demonstrated that enhanced generation of ROS by mitochondria increases BACE1 activity and consequently promotes amyloidogenic APP processing and A*β* overproduction [88]. This is consistent with other studies showing that hydrogen peroxide- (H_2_O_2_-) induced oxidative stress increases the activity of *BACE1* promoter in human embryonic kidney (HEK) cells [89] and results in elevated levels of secreted A*β*42 in differentiated SH-SY5Y neuroblastoma cell line [90]. Of note, intracellular A*β*42 accumulation is also observed upon H_2_O_2_ treatment, but this is apparent only during the first hour of the stimulation and decreases to the baseline later during the time course [90]. A*β* generation in the oxidative conditions might be also affected by changes in the function of the PS1/*γ*-secretase complex. Injection of 4-HNE or 4,4′-dithiodipyridine (DTDP) into the mouse brain has been reported to induce pathogenic shift in the arrangement of PS1 subdomains within the *γ*-secretase complex, resulting in enhanced generation of longer, more prone to aggregation A*β* species (A*β*42), relative to the shorter forms (A*β*40) [91, 92]. Other studies have suggested that the PS1 conformational change might be a consequence of 4-HNE modification of one of the members of the *γ*-secretase complex—nicastrin—in the oxidative environment [93].

Importantly, it has been reported that not only ROS can modulate A*β* production/secretion but also A*β* can reciprocally promote excessive ROS generation in a vicious cycle. A*β* overproduction, achieved by APP overexpression, has been shown to enhance ROS generation, reduce the respiratory control ratio (RCR), and diminish ATP production, suggestive of the existence of a positive ROS→A*β* feedback loop [88]. Exposure of neurons to oligomeric A*β* leads to enhanced ROS production, probably through a mechanism involving stimulation of N-methyl-D-aspartic acid (NMDA) receptors and consequent activation of NOX. ROS derived from NOX may lead to the activation of kinases, including ERK1/2, downstream activation of calcium-dependent phospholipase A2 (cPLA_2_), release of arachidonic acid, and perturbations in the membrane phospholipids [94–96].

The detrimental role of APP/A*β* in the induction of oxidative stress has been also reported in several *in vivo* studies [97]. For example, a transgenic AD mouse model, harboring APP KM670/671NL and APP V717F mutations, has been reported to present elevated levels of protein and lipid oxidation markers, such as protein carbonyls, 3-NT, and 4-HNE. Interestingly, the observed oxidative damage appears to be dependent on the methionine 35 in the A*β* peptide [98, 99]. Furthermore, intracranial imaging of APP KM670/671NL, PS1dE9 mouse brains has revealed that senile plaques are directly responsible for ROS generation [100].

## 4. Mitochondrial Impairments in AD Brain

There are compelling evidences that aberrant mitochondrial structure and function are important hallmarks of AD pathology. The expression and activity of mitochondrial enzymes, such as cytochrome c oxidase (COX), *α*-ketoglutarate dehydrogenase complex, and pyruvate dehydrogenase complex, are reduced in AD [101, 102]. Moreover, AD brain mitochondria present diminished membrane potential and increased permeability, and produce excess ROS which lead to protein, lipid, and nucleic acid damage. Furthermore, abnormal mitochondrial structure, decreased anterograde movement, and compromised mitochondrial functions have been described in neurons from an APP transgenic AD mouse model [103]. Finally, increased autophagy of mitochondria [104, 105] and altered mitochondrial structure have been observed in AD brains [106].

Although mitochondrial impairments are clearly associated with AD pathology, it is an ongoing debate whether they cause or result from an altered A*β* and tau biology. Several studies imply that mitochondrial cascade hypothesis may explain the etiology of late-onset, SAD. This hypothesis states that mitochondrial dysfunctions are the primary events leading to aberrant APP processing and A*β* deposition [28, 107]. In line with this hypothesis, chemical inhibition of oxidative energy metabolism has been reported to modulate APP processing in PC12 cells [108] and in COS cell line stably expressing APP [109, 110]. Moreover, combination of FDG- and PiB-PET imaging has demonstrated that brain hypometabolism is detected before A*β* deposition in cognitively normal individuals at risk for developing AD [111].

On the other hand, it is also possible that early alterations in A*β* biology trigger mitochondrial pathology. It has been reported that A*β* peptides interact directly with A*β*-binding alcohol dehydrogenase (ABAD) in mitochondria, and via this interaction, A*β* may cause leakage of ROS and mitochondrial dysfunctions [112]. Moreover, A*β* has been demonstrated to detrimentally impact mitochondrial enzymes, which contain iron-sulphur center, inhibit complex I and IV, reduce ATP levels, and induce mitochondrial depolarization [113–118]. A*β* may also increase the expression of heme oxygenase 1 (HO-1) enzyme which may secondarily compromise mitochondrial integrity [119], and A*β* plaques have been proposed to be a source of toxicity that induces severe mitochondrial structural and functional abnormalities *in vivo* in the mouse brain [120]. In addition, several studies indicate that APP and A*β* might affect fussion and fission of mitochondria. Elevated expression of mitochondrial fission genes, encoding dynamin-related protein 1 (Drp1) and fission 1 (Fis1), and reduced expression of the fussion ones, encoding mitofusin 1 (Mfn1), mitofusin 2 (Mfn2), optic atrophy 1 (Opa1), and Tomm40, have been detected in AD brains, fibroblasts from AD patients, neurons isolated from APP transgenic mice, and cell lines overexpressing APP [121–123]. Moreover, treatments of neurons with A*β* peptides have been shown to affect mitochondrion movement [124]. All these may result in structural changes of mitochondria, decreased clearance of defective mitochondria, and enhanced neurodegeneration.

Not only A*β* but also tau might lead to mitochondrial pathology. Tau protein has been shown to play an important role in the trafficking of organelles, including mitochondria, and this tau function might be compromised in neurons in AD. In line with that, overexpression of tau in CHO and N2A cell lines as well as in neurons results in subcellular misdistribution of mitochondria, potentially via alterations in Drp1 mitochondrial location [125, 126], and genetic reduction of tau rescues aberrant mitochondrial trafficking in APP transgenic neurons [127]. Furthermore, it has been demonstrated that A*β*-triggered hyperphosphorylation of tau leads to its detachment from microtubules and abnormal mitochondria trafficking [128]. Finally, N-terminal fragment of tau has been reported to colocalize with adenine nucleotide translocator 1 (ANT1) in AD mitochondria, impair ANT function, and consequently compromise mitochondrial oxidative phosphorylation [129, 130].

Overall, these data suggest that oxidative stress is an early factor in AD pathogenesis that can lead to mitochondrial dysfunction, cell signaling impairments, and increased production of A*β*. In turn, A*β* overproduction, oligomerization, and aggregation reciprocally augment the oxidative stress. Once the positive feedback loop between ROS production and A*β* is initiated, it gradually enhances and accelerates the brain pathology.

## 5. Compromised Oxidative Stress Defense Mechanisms in AD Brain

On the opposite site of the ROS generation machinery stands the entire network of the oxidative stress defense mechanisms, developed to detect and control diverse forms of the oxidative stress. These include glutathione redox cycle, heat shock pathways, hemoxygenase and thioredoxin (Thx) systems, and superoxide peroxidases. Redox-regulated proteins, being part of these systems, are encoded by vitagenes, proposed to assure longevity [131, 132].

One of the most prevalent antioxidants in the cell is glutathione, which exists in a dynamic pool of thiol-reduced (GSH) and disulfide-oxidized states. GSH can react with free radicals either independently or in the reaction catalyzed by glutathione peroxidase (GPx) to form glutathione disulfide (GSSG), which can be converted back to the reduced state by glutathione reductase (GR). Alternatively, glutathione S-transferases (GST) can catalyze a reaction between GSH and nucleophilic compounds that react with thiols in proteins. Considering the antioxidant properties of GSH and its depletion under oxidative stress conditions, GSH/GSSG ratio is frequently used as an indicator of cell redox potential and oxidative stress [133, 134] and has been also analyzed to assess the oxidative stress in AD. Postmortem analysis of the human brains revealed decreased GSH levels and increased GSSG amount, resulting in diminished GSH/GSSG ratio, in postmitochondrial supernatant (PMS), mitochondrial, and synaptosomal fractions obtained from frontal cortices of MCI, as well as mild/moderate and severe AD patients compared to controls [135, 136]. Similar results have been obtained with the application of proton magnetic resonance spectroscopy for *in vivo* GSH analysis in human subjects. Importantly, the reduction in GSH has been detected selectively in the brain regions affected by AD pathology, such as frontal cortex and hippocampus, but not in the cerebellum, and correlated with the extent of cognitive impairments, assessed with Mini-Mental State Examination (MMSE) and clinical dementia rate (CDR) scores [137]. In addition to the altered GSH/GSSG balance, a trend towards decreased activity of GR, GPx, and GST enzymes has been detected, with statistically significant changes recorded in mitochondrial fraction from AD versus controls and in the synaptosomal fraction from both MCI and AD patients versus control individuals. Interestingly, the magnitude of the changes in the glutathione redox cycle seems to correspond with the disease severity [135, 137].

Another important antioxidant enzymes are superoxide dismutase (SOD) and catalase (Cat), which catalyze the disproportionation of superoxide to molecular oxygen and peroxide and the conversion of H_2_O_2_ to water and oxygen, respectively. The activity of these enzymes has been reported to be reduced through the entire AD continuum, from MCI to severely cognitively impaired patients, indicative of compromised oxidative stress defense mechanisms already early in the disease [135]. Of note, Karelson et al. reported an increase in SOD and Cat activities, selectively in temporal inferior, but not in the frontal inferior, sensory postcentral, and occipital cortices in AD versus control brains [136]. In addition, a trend towards reduced mRNA expression of peptide methionine sulfoxide reductase (MsrA), an enzyme converting methionine sulfoxide to methionine, and significantly diminished MsrA activity, to a much greater extent, has been detected in AD-affected brain regions, such as hippocampus, superior and middle temporal gyri, and inferior parietal lobule [138]. In addition, reduced levels of Thx, a potent ROS scavenger, have been observed in amygdala, hippocampus, and superior and middle temporal gyri in AD [139]. Reduced transcription of detoxifying genes and diminished expression of antioxidant enzymes are in line with decreased levels of nuclear factor (erythroid-derived 2)-like 2 (Nrf2), a transcription factor activated upon exposure to oxidative stress [140], in the nucleus of hippocampal neurons in human AD brains [141].

On the other hand, increased protein levels and/or activity of enzymes, which are induced as protective mechanisms against oxidative stress, have been reported. Thx reductase activity appears to be elevated in amygdala and cerebellum in AD versus controls [139]. In addition, increased HO-1 protein expression has been reported in AD, with significant changes observed only in neocortex and hippocampus but not in substantia nigra. Chronic upregulation of HO-1 may exacerbate the degenerative process by promoting pathological iron deposition and oxidative mitochondrial damage [142]. Interestingly, detailed immunofluorescence analysis of the HO-1 localization has revealed that the expression of this enzyme is elevated both in neurons and glial fibrillary acidic protein- (GFAP-) positive astrocytes and that senile plaques and neurofibrillary tangles are immunopositive for HO-1 [143]. Importantly, the percentage of double GFAP and HO-1-positive astrocytes is much higher in temporal cortex and hippocampus of MCI and AD patients compared to controls and shows a negative correlation with global cognitive performance and a positive one with AD pathology, with the latter apparent only in the temporal cortex [144]. The induction of the expression of the protective enzymes has been proposed to be a result of a chronic exposure to high levels of oxidative stress in AD [143, 144]. Overall, the described studies indicate that AD is associated with unsuccessful cellular attempts to induce oxidative stress defense mechanisms and to compensate for the oxidative damage.

## 6. Oxidant/Antioxidant Imbalance as a Therapeutic Target in AD

Compelling evidence presenting disrupted oxidant/antioxidant balance in AD led to the formulation of a hypothesis that compounds scavenging free radicals, and/or boosting oxidative stress defense mechanisms might provide therapeutic benefits in AD. Therefore, several antioxidants, such as N-acetylcysteine, curcumin, resveratrol, vitamin E, ferulic acid, coenzyme Q (CoQ), selenium, and melatonin, have been tested for their potential to preserve or improve cognitive performance in healthy individuals, MCI, and AD, as reviewed in [145–147]. However, despite potent effects of these compounds on cellular oxidative status and/or A*β* pathology *in vitro* and *in vivo*, the convincing evidence of their therapeutic potential in humans is lacking.

Recently, Mazzanti et al. reviewed a number of published and ongoing clinical trials with the use of curcumin and resveratrol for AD therapy [148, 149]. These compounds are known for their potent antioxidant activity *in vitro* and *in vivo*. They have a potential to scavenge nitric oxide (NO) and hydroxyl radicals, increase brain glutathione content and HO-1 expression, activate sirtuins, and prevent A*β*-mediated ROS accumulation [150]. Despite all these promising data, clinical trials demonstrated that the efficacy of these compounds in preserving or restoring cognitive functions in humans, both in healthy individuals as well as in clinical AD patients, is limited [148].

Similarly, there is no convincing evidence that supplementation with vitamin E, known to protect lipids from peroxidation, can provide therapeutic benefits in healthy individuals, MCI, and AD. Although few clinical trials have demonstrated some delay in the cognitive decline upon vitamin E administration, others have not shown any associations of the use of this compound with reduced cognitive deterioration, or even reported detrimental effects of vitamin E on neuropsychological functions of MCI and AD patients [151, 152]. Similarly, negative data have been reported in the trial of a combination of vitamin E with other antioxidants, such as vitamin C and *α*-lipoic acid. Despite the decrease in F2-isoprostanes in the CSF, consistent with antioxidant potential, no change in other CSF biomarkers, such as A*β*, tau, or phospho-tau, and more rapid MMSE score decline have been found in the treatment versus the placebo group [153]. In the latter study, CoQ supplementation in AD patients has been also evaluated. Unfortunately, the results have been also negative [153]. Similarly, no associations between the long-term use of antioxidants, such as vitamin E and selenium, and dementia incidence in healthy individuals were found in the large-scale primary prevention trial [154].

On the other hand, other large-scale epidemiological studies have given more promising results, suggesting that eating food reach in antioxidant supplements, beta carotene, and vitamins C and E is associated with lower risk of AD dementia [155, 156]. More hopeful data stem also from the clinical trials with glutathione precursor, N-acetylcysteine, and melatonin. It has been demonstrated that administration of N-acetylcysteine might provide modest cognitive benefits in AD patients [147]. Similarly, treatment with melatonin seems to improve sleep quality and cognitive performance in MCI patients. However, more research is needed to provide evidence of its efficacy in AD patients [157]. A recent meta-analysis of randomized double-blind clinical trials of melatonin in AD has demonstrated that the compound improves total sleep at night but has no effect on the MMSE score in AD [158].

Furthermore, as previously highlighted, alterations in mitochondrial bioenergetics, defects in mitochondrial dynamics, and ROS-mediated mitochondria damage play a key role in AD pathogenesis. Therefore, strategies that target mitochondrial dysfunctions, enhance mitochondrial bioenergetics, or reverse oxidative stress may serve as novel AD therapeutics. One of such therapeutics might be CoQ10, known also as an ubiquinone, which acts as an important cofactor of the ETC. Both *in vitro* and *in vivo* studies have demonstrated the neuroprotective potential of CoQ10 in AD [159, 160]. It has been shown that this lipophilic antioxidant compound improves cognitive functions, facilitates ATP synthesis, and upregulates mitochondrial function [161]. Furthermore, it is well documented that CoQ10 supplementation significantly increases brain CoQ10 amount, providing protection from free radical-mediated oxidative damage to biomolecules [162]. An analog of CoQ10 is idebenone. This compound consists of short chains of isoprene units, crosses blood-brain barrier easily, is well tolerated in humans, possesses good antioxidant properties, and provides neuroprotection against A*β*-induced neurotoxicity [163, 164]. Another potent antioxidant, acting as an effective inhibitor of mitochondrial permeability transition pore (mPTP), is creatine [165]. It exerts neuroprotective potential in different neurodegenerative diseases, including AD. Creatine supplementation has been shown to protect against neuronal death caused by NMDA, malonate, A*β*, and ibotenic acid [166].

In addition, due to the importance of mitochondrial dysfunctions in the pathogenesis of various disorders, scientists have focused on the engineering of therapeutic molecules that could accumulate in mitochondria. One of such compounds is MitoQ, which is the most widely used mitochondria-targeting antioxidant. MitoQ exhibits neuroprotection via scavenging peroxynitrite and superoxide and consequently protecting mitochondria against lipid peroxidation [167]. Another antioxidant acting in a similar manner is MitoVitE, also known as mitotocopherol, which is triphenylphosphonium (TPP) conjugated to *α*-tocopherol moiety of vitamin E via two-carbon chain. It also protects mitochondria from oxidative stress via inhibition of lipid peroxidation [168, 169]. Another used TPP derivative is MitoTEMPOL, which acts by accepting an electron from hydroxylamine. Moreover, MitoTEMPOL functions as a SOD mimetic, whose role is to convert superoxide molecules into water to detoxify ferrous iron into ferric iron. Its beneficial effects against mitochondrial dysfunction and mitochondria-mediated oxidative stress were shown in an *in vitro* study [170].

The question arises why, despite the clear implication of oxidative stress in AD pathology, and beneficial action of ROS targeting therapeutics *in vitro* and in mouse models, drugs targeting impaired oxidant/antioxidant balance quite often fail to provide benefits in clinical trials. It is possible that due to the physiological importance of moderate ROS levels and redox signaling, high doses of antioxidants, as often administered in clinical trials, are detrimental rather than beneficial for the cell physiology [39, 171]. In line with that, administration of high vitamin E doses has been reported to increase mortality [172].

Furthermore, the tested antioxidant therapeutics might present poor bioavailability and efficiency in reaching their targets due to their low solubility and absorption, together with a rapid metabolism and elimination [150, 173]. These limitations might be overcome by the development of novel delivery systems, such as adjuvants, nanoparticles, liposomes, micelles, phospholipid complexes, and nanogels. Interestingly, selenium, melanin, and cerium oxide-nanoparticles have been reported to reduce ROS levels and boost oxidative stress defense mechanisms *in vitro* and *in vivo*. Similarly, some of the preparations improving the bioavailability of curcumin and resveratrol have been reported to present therapeutic benefits, as reviewed in [148]. This raises a potential that such compounds might become novel therapeutics for oxidative stress-related disorders, including AD [174–178].

Another reason might be the inappropriate design of the clinical trials [148, 179, 180]. The number of recruited patients is usually relatively small, and the duration of the follow-up is inadequately short, preventing the possibility to detect potential improvements in cognitive functions. Moreover, the treatments are often administered to patients at the advanced AD stage with clearly developed clinical symptoms. It needs to be noted that molecular pathogenic processes in the brain precede by decades the onset of symptomatic cognitive decline. In symptomatic AD or even in MCI, the pathological changes, such as A*β* accumulation and synapse loss, are already evident and probably cannot be reversed by the use of antioxidants [1, 181]. Therefore, the therapeutics should be administered earlier, before the substantial synaptic/neuronal loss. Unfortunately, the AD field suffers from the lack of diagnostic tools which would allow the detection of the disease at its presymptomatic phase. This leads to the inadequate recruitment of patients for clinical trials and might be improved by the discovery of novel easily accessible, optimally blood-based biomarkers allowing not only the diagnosis of AD at the preclinical phase but also sensitive biomarker-based monitoring of the therapeutic efficacy of the administered drugs.

## 7. Diagnostic Prospects Based on Oxidative Stress Biomarkers in Blood Cells

The AD diagnostics critically needs novel biomarkers for early AD detection. These have to be cost-effective and located in easily available diagnostic tissues, which are not the case for the currently used CSF or brain imaging biomarkers, reviewed in [182]. Among the main advantages of the blood-based biomarkers are their lower risk and discomfort for patients and thus increased applicability in an ageing population. Blood-based biomarkers for early AD can be employed as a first step in large-scale screening to recruit patients who should undergo further, more sophisticated and costly testing. Moreover, they are adequate for repeated measures and hence could be useful for monitoring therapeutic outcomes in longitudinal studies. However, so far, no blood-based biomarkers have been accepted for AD diagnostics.

Novel prospects for identification of AD biomarkers in blood have emerged recently based on systemic presentation of oxidative stress. Namely, several studies have demonstrated that the imbalance in oxidative stress and antioxidant defense occurring in the brain is reflected in peripheral cells and in particular in easily accessible blood cells that could be used for early disease diagnostics and therapy monitoring, as reviewed in [59, 60, 182, 183]. Accordingly, it has been reported that ROS levels are elevated in AD lymphocytes and platelets compared to controls [184, 185]. This increase seems to correspond to higher levels of oxidative damage markers, such as nitric oxide synthase 2 (NOS-2), 3-NT, protein carbonyls, and 4-HNE [186]. Furthermore, fluorescence spectroscopy analysis of lipofuscin-like pigments (LFPs), end products of lipid peroxidation, has demonstrated increased LFP levels in AD erythrocytes compared to controls [187]. Of note, other studies have suggested that the increase in 3-NT and 4-HNE is specific only to lymphocytes from FAD patients and is not marked in the sporadic cases [188], suggesting that oxidative stress is associated with early A*β* overproduction.

Not only proteins and lipids but also nucleic acids can undergo oxidative damage. Consistently, application of high pressure liquid chromatography (HPLC) for the analysis of 8OHdG, a DNA oxidation by-product, has revealed elevation of this marker in AD versus control lymphocytes [189, 190]. Furthermore, using comet assay, modified with oxidative lesion-specific endonucleases, it has been demonstrated that AD lymphocytes contain significantly elevated levels of oxidized purines [191, 192]. All these data support the hypothesis that oxidative damage to proteins and nucleic acids in AD is a systemic event that manifests in peripheral cells in blood.

## 8. Mitochondrial Dysfunctions in Blood Cells as Potential AD Biomarkers

Particular emphasis in the search for novel oxidative stress-related blood-based biomarkers has been placed on mitochondria since they are the main source of oxidative stress, and their impairments may result in the leakage of superoxide radicals and consequent increased generation of reactive nitrogen and oxygen species [193]. This resulted in the report of the elevation of the protein-bound 4-HNE and 3-NT in the mitochondria isolated from lymphocytes from MCI patients, with the levels of protein-bound 4-HNE showing a significant negative correlation with the MMSE score [194]. Furthermore, several mitochondrial proteins, grouped in four categories, cell signaling, structural, cellular energetics, and cellular defense, that are altered between lymphocytes from control versus MCI versus AD patients have been identified. This points towards particular pathways that might be responsible for the initiation of cognitive decline and for the later transition from MCI to AD [195]. Another evidence of the oxidative damage to the mitochondria in blood lymphocytes in AD has been provided by the analysis of the mitochondrial aconitase 2 (ACO2), which is a Krebs cycle enzyme sensitive to free radical-mediated damage, due to the presence of an iron-sulphur group [196]. It has been demonstrated that ACO2 expression and activity are significantly lower in AD and MCI lymphocytes compared to controls and that these changes correlate with MMSE score [197].

In addition, assuming that impaired mitochondrial energetics may be causative of the excess generation of ROS, several groups have analyzed the activity of mitochondrial complexes in peripheral cells/elements, that is, lymphocytes and platelets from MCI and AD patients, but these studies resulted in contradicting data. Molina et al. reported no changes in the activity of the respiratory chain enzymes in the mitochondria isolated from lymphocytes from AD patients and control individuals [198], while later reports have shown decreased activity of complexes III and IV in the mitochondria isolated from AD platelets and decline in the activity of complex IV in these from MCI patients [199]. On the other hand, Feldhaus et al. observed increased activity of the complex II and complex IV in AD lymphocytes [200]. Despite the discrepancies, these potential impairments in the function of the respective mitochondrial complexes may manifest in the altered oxidative phosphorylation (OXPHOS), that is, mitochondrial respiration capacity, which represents the entire electron transport chain, comprised of the four complexes and F1F0ATP synthase. Indeed, basal level of respiration, total OXPHOS capacity, and RCR, indicative of mitochondria coupling state, are diminished in AD compared to control lymphocytes [201]. All these studies imply that mitochondrial energetics and oxygen consumption show impairments already early in the disease. This might lead to increased ROS generation and consequent cascade of oxidative damage at the later stages.

## 9. Reflection of Impaired Oxidative Stress Defense Mechanisms in Blood Cells

The alterations of the oxidative stress defense mechanisms have been also detected in peripheral tissues, including blood cells, supporting the hypothesis of the systemic nature of the disease (Table 1).

Reduced GSH levels with unchanged GSSG amount in lymphoblasts from FAD but not from SAD patients have been detected [202]. More recent studies have demonstrated a reduction in GSH and an increase in the GSSG in MCI and SAD lymphocytes and plasma [186, 203, 204] and a correlation between plasma GSH/GSSG ratio and MMSE score [205]. Finally, it has been reported that both FAD and SAD lymphocytes present reduced GR activity [188]. However, it might be possible that these changes represent only full-blown AD clinical pathology since the analysis of samples from young healthy individuals at risk of developing AD, due to the carriage of at least one ApoE4 allele, has demonstrated reduced GSSG levels in whole blood and increased amount of GPx and glutamylcysteine ligase (GCL) in lymphocytes when compared to the ApoE3/ApoE3 controls. These data are indicative of the existence of reductive rather than oxidative stress at the presymptomatic state of disease. The situation is reversed in MCI and clinical AD, potentially due to the exhaustion of oxidative stress defense mechanisms [203, 206].

In addition to GSH, other markers of altered oxidative stress defense mechanisms have been also studied and found to be changed in AD versus control peripheral blood samples. Levels and/or activity of sirtuin-1 (SIRT-1), Thx, HO-1, Hsp60, Hsp72, and Thx reductase (ThxR) are elevated in plasma and/or lymphocytes from AD versus control samples [186, 207]. Moreover, mRNA levels of CuZnSOD are higher in lymphocytes from AD patients when compared to healthy controls as well as Parkinson's disease patients, suggestive of disease specificity [208]. These changes probably represent cellular attempts to overcome consequences and survive constant exposure to high levels of ROS and are consistent with the activation of transcription factors, such as Nrf-2, and the phosphorylation of which at the serine 40 has been reported to be significantly increased in peripheral mononuclear blood cells (PBMC) from MCI patients and present a modest, although nonsignificant, elevation at the later stages of the disease [209]. However, the defense mechanisms seem to be not entirely functional in AD cells. Despite the increased CuZnSOD mRNA levels [208], no change in CuZnSOD and MnSOD protein levels and/or diminished Ec-CuZnSOD activity in MCI, SAD, and FAD lymphocytes [58, 188] and in SAD erythrocytes [210] has been reported. Of note, there is some inconsistency in the reports of the changes of SOD expression in different studies. Opposed to the other reports, Mota et al. demonstrated that the protein levels of SOD are significantly diminished in PBMC from MCI patients and show a trend towards an elevation in PBMC from mild AD patients [209]. Inconsistencies are also present between the reports on the HO-1 expression. While some studies demonstrated increased protein levels of HO-1 in AD lymphocytes [186], others have shown decreased HO-1 mRNA and protein levels in lymphocytes, plasma, choroid plexus, and CSF from early AD and from some amnestic MCI patients [211, 212] and augmented activity of HO-1 suppressor (HOS) in AD plasma [213]. The inconsistencies might be due to different criteria used for patient selection, population variabilities, and/or various experimental strategies applied in the studies. Nevertheless, levels of other stress defense mechanisms such as those involving mitochondrial uncoupling protein 1 (UCP1), known to reduce superoxide and H_2_O_2_ generation, are also diminished in AD plasma [207]. Consequently, the impaired oxidative stress defense mechanisms may yield cells from AD patients more vulnerable to H_2_O_2_-induced oxidative stress-triggered cell death. In line with that, AD lymphocytes cannot induce CuZnSOD mRNA expression upon H_2_O_2_ stimulation and their vulnerability to the cell death correlates with dementia severity [208, 214, 215].

Yet another evidence of the compromised defense mechanisms in AD stems from the observation of reduced total antioxidant power, a cumulative measure of the amount of all plasma antioxidants, such as vitamin E, uric acid, vitamin C, bilirubin, thiols, and glutathione, in AD plasma versus Huntington's disease control [216]. Further detailed analyses demonstrated diminished levels of vitamin E (tocopherol) in plasma from MCI and mild AD patients compared to controls [204, 217]. In addition, amounts of another micronutrient, selenium, essential component of selenocysteine and a cofactor of GPx and ThxR, important in cell defense against oxidative stress, have been shown to be reduced in plasma and erythrocytes from AD patients [210] and to correlate with cognitive decline, as evidenced in a 9-year longitudinal study in patients between 60 and 71 years of age [218].

Altogether, the above findings are supportive of the hypothesis that not only ROS generation but also the cellular oxidative stress defense mechanisms are compromised in the brain as well as in peripheral tissues in AD. The molecular alterations in blood cells that could be detected already at the preclinical AD stage might be further tested as potential easily accessible novel biomarkers for early AD.

## 10. Conclusions and Future Perspectives

Several evidences indicate that oxidative stress and impaired oxidative stress defense are among the main early causes of age-related neurodegeneration in AD brain. Moreover, the progression of AD seems to be associated with an interplay between oxidative stress and A*β* in a vicious cycle. Consequently, several antioxidants have been tested for their potential to preserve or improve cognitive performance in MCI and AD patients but most of them failed to provide clinical benefits. The failures of the clinical trials might be attributed to the administration of incorrect doses and/or poor bioavailability of the drugs. The latter could be improved by the development of novel nanotechnologies. Furthermore, the disappointing results of the clinical trials might be related to the inappropriate recruitment of patients, administration of drugs at the advanced AD stages, and too short follow-ups to detect clinical benefits. The selection of patients and monitoring of the clinical trials can be improved by the design of novel noninvasive biomarker-based tools allowing the detection of the disease at the presymptomatic stage and sensitive long-term monitoring of therapeutic efficacies.

Importantly, several studies have demonstrated that the oxidant/antioxidant imbalance in early AD is reflected in easily accessible blood cells. This opens new avenues for the development of noninvasive, blood-based biomarkers that could overcome the limitation of the currently employed CSF assays and brain imaging.

## Figures and Tables

**Table 1:** Impaired oxidative stress defense mechanisms in the brain and peripheral blood in AD.

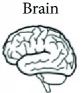	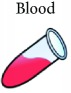
GSH ↓, GSSG ↑, GSH/GSSG ↓ [135, 136]	GSH ↓, GSSG ↓↑ [186, 202–204]
GR activity ↓, GPX activity ↓, GST activity ↓ [135, 137]	GR activity ↓ [188] GPX protein ↑, GCL protein ↑ [203, 206]
SOD activity ↓↑ [135, 136]	SOD mRNA ↑, SOD activity ↓ [58, 188, 209]
Thx protein ↓, ThxR protein ↑ [139]	Thx protein ↑, ThxR protein ↑ [186, 207]
HO-1 protein ↑ [143, 144]	HO-1 protein ↑, HO-1 activity ↑ [186, 207]
Nrf-2 in nucleus ↓ [141]	P-Nrf2 ↑ [209]
Cat activity ↓↑ [135, 136]	Total antioxidant status ↓ [216]
MsrA mRNA ↓ [138]	SIRT-1 protein ↑ [186, 207]
	HSP60 protein ↑, HSP70 protein ↑ [186, 207]
	UCP1 protein ↓ [207]
	Vitamin E ↓ [204, 217]
	Selenium ↓ [218]

The table summarizes the changes in the oxidative stress defense mechanisms reported in the brain and blood from MCI and/or AD patients relative to healthy controls; the corresponding references are given; arrows indicate direction of changes in protein levels or activities, described in detail in the text. AD: Alzheimer's disease; MCI: mild cognitive impairment; Cat: catalyse, GCL: glutamylcysteine ligase; GSH: reduced glutathione; GSSG: oxidized glutathione; GPx: glutathione peroxidase; GR: glutathione reductase; GST: glutathione S-transferase; HO-1: heme oxygenase 1; Hsp: heat shock protein; MsrA: methionine sulfoxide reductase A; Nrf-2: nuclear factor (erythroid-derived 2)-like 2; SIRT-1: sirtuin 1; SOD: superoxide dismutase; Thx: thioredoxin; ThxR: thioredoxin reductase; UCP1: uncoupling protein 1.
